# Development and Face and Content Validation of Augmedicine: Ultrasound—An Extended Reality Application for Ultrasound Training

**DOI:** 10.1002/ajum.70019

**Published:** 2025-08-21

**Authors:** Tessa A. Mulder, Robin Jacobs, Manon S. Zuurmond, Adel Qaddoumi, Martijn P. Bauer, Alexandr Srámek, Beerend P. Hierck, Alexandra M. J. Langers

**Affiliations:** ^1^ Department of Internal Medicine Leiden University Medical Center Leiden the Netherlands; ^2^ Leiden Learning and Innovation Center Leiden University The Hague the Netherlands; ^3^ Department of Radiology Leiden University Medical Center Leiden the Netherlands; ^4^ Center for Innovation in Medical Education Leiden University Medical Center Leiden the Netherlands; ^5^ Department of Clinical Sciences‐Anatomy and Physiology Veterinary Medicine Faculty, Utrecht University Utrecht the Netherlands; ^6^ Department of Gastroenterology and Hepatology Leiden University Medical Center Leiden the Netherlands

**Keywords:** 3‐dimensional visualisation, echocardiography, extended reality, point‐of‐care ultrasound, simulation‐based education

## Abstract

**Objectives:**

Point‐of‐care ultrasound (POCUS) is a bedside imaging technique increasingly taught to physicians and medical students. Beginners often face challenges with probe orientation and hand–eye coordination. Extended reality (XR) can enhance POCUS education by projecting a 3D anatomical model onto a phantom or patient, providing unlimited training and direct feedback on probe orientation and image acquisition. We developed Augmedicine: Ultrasound, an XR application for training point‐of‐care echocardiography. This study evaluates the face and content validity of the application.

**Methods:**

Three groups (novices, intermediates and experts in ultrasonography) were invited to complete tasks using the application. Following these tasks, all participants filled out a questionnaire to rate realism, didactic value, and usability of the application using a 5‐point Likert‐scale and open‐ended questions.

**Results:**

A total of 61 participants (43 novices, 10 intermediates, 8 experts) completed the evaluation. The application was recognised as a valuable tool for novice training, with 95% indicating that it is useful to enhance spatial understanding and 77% for improving hand–eye coordination. Anatomical accuracy was rated positively by 90% of participants, and 69% found the application easy to use. Experts highlighted its potential for expansion, such as inclusion of procedural skills and pathology training.

**Conclusion:**

Our XR ultrasound application demonstrated face and content validity for echocardiography training. It holds promise as a complementary tool to traditional methods, supporting novice learners by improving spatial understanding and reducing reliance on instructors and test subjects. Future iterations will address technical optimisations, improve realism, and evaluate the application's learning effect.

**Summary**

- Point‐of‐care ultrasound (POCUS) is an essential bedside tool for physicians, but learning to perform and interpret ultrasound images requires significant practice.
- Our study introduces Augmedicine: Ultrasound, an Extended Reality (XR) application designed to improve POCUS training.
- By projecting a 3D anatomical model onto a phantom, it provides real‐time guidance on probe positioning and image acquisition, helping novices develop critical spatial understanding.
- Through validation with medical students, residents, and experts, we demonstrate its realism, usability, and educational value.
- This application has the potential to enhance ultrasound education by offering unlimited, self‐directed training, reducing reliance on instructors and volunteer patients.

## 1 Introduction

Point‐of‐care ultrasound (POCUS) is a bedside imaging technique focused on addressing specific diagnostic questions. Unlike other imaging modalities, POCUS heavily relies on the operator's skills. Traditionally reserved for certain medical specialists, ultrasound in general and POCUS in particular are now being adopted by a growing number of physicians and incorporated into medical school curricula [1]. This expanded adoption presents significant challenges in scaling training effectively while maintaining quality [2].

Training in POCUS is resource intensive. It demands dedicated instructors who must provide close supervision and immediate feedback during hands‐on sessions. Additionally, learners often require volunteers or patients to practise on, which creates logistical challenges [3, 4]. These limitations are compounded by the steep learning curve for mastering critical skills, including 3D anatomical thinking, probe orientation, and hand–eye coordination [4].

Increasingly, 3D simulation software and extended reality (XR) applications—including Virtual Reality (VR), Augmented Reality (AR), and Mixed Reality—are being developed as a solution to these challenges [5, 6, 7, 8]. XR enriches real‐world environments by adding interactive 3‐Dimensional (3D) projections, offering unique opportunities for active learning in authentic yet controlled learning environments, without the restraints of traditional training. Trainees benefit from repetitive, self‐paced learning, which is enriched with automated personalised feedback, while maintaining the possibility of collaboration with peers and instructors. Its use in ultrasound education is especially promising, as XR allows for the projection of a 3D anatomical model inside a phantom or patient, providing immediate feedback on probe orientation and corresponding ultrasound images [9]. For example, Ebner et al. [10] showed that their AR application improves the accuracy of kidney measurements during an ultrasound task. An additional benefit lies in the conservation of teachers and volunteers for hands‐on practice when part of the training can be done with the AR application. This proves particularly valuable in situations where large numbers of students need to be trained.

Building on these advancements, we developed an XR application specifically focused on echocardiography training. This application aims to improve the learning process by combining 3D anatomical visualisation with ultrasound image generation, providing users with direct feedback on their probe positioning and the resulting ultrasound views. Echocardiography, being the most widely utilised POCUS application [11], provides a range of difficulty levels, from basic image generation to advanced tasks like valve function assessment. This makes it suitable for learners of varying expertise while also offering objective tracking of skill development over time. Additionally, cardiac anatomy and physiology present unique challenges for novices, making echocardiography an ideal focus for a tool designed to improve anatomical understanding and probe‐handling skills.

This article describes the development of our XR‐based application and explores its potential to enhance POCUS education. We gathered feedback from different stakeholders to refine the application and assess its educational value [12]. Specifically, we evaluated the face and content validity of the application. Face validity refers to the degree to which a simulator accurately represents the skills required in a real‐world environment, specifically addressing how realistic the simulator appears for training in POCUS. Content validity evaluates how useful the simulator appears as a tool for learning relevant skills, such as mastering probe‐handling techniques and obtaining accurate cardiac ultrasound images [6, 13]. This study aims to assess both the realism (face validity) and the educational value (content validity) of our XR application, based on feedback from a diverse group of stakeholders.

## 2 Materials and Methods

### 2.1 The Application

#### 2.1.1 The Virtual 3D Ultrasound Environment

The Augmedicine: Ultrasound application provides an interactive virtual environment for ultrasound training. When users put on the HoloLens 2 headset (Microsoft Corporation, 2023, https://www.microsoft.com/hololens), they remain aware of their physical surroundings while virtual elements are added to their environments. They see a 3‐dimensional virtual patient in the supine position and a virtual ultrasound probe projected onto their phone, which functions as both the probe and the user interface. A virtual beam extending from the virtual probe represents ultrasound waves and their interaction with the virtual patient. This is accompanied by a virtual ultrasound screen that displays real‐time images based on the virtual patient and probe movements. These images were dynamically generated by the application. Care was taken to simulate natural ultrasound images as well as possible.

**FIGURE 1:**
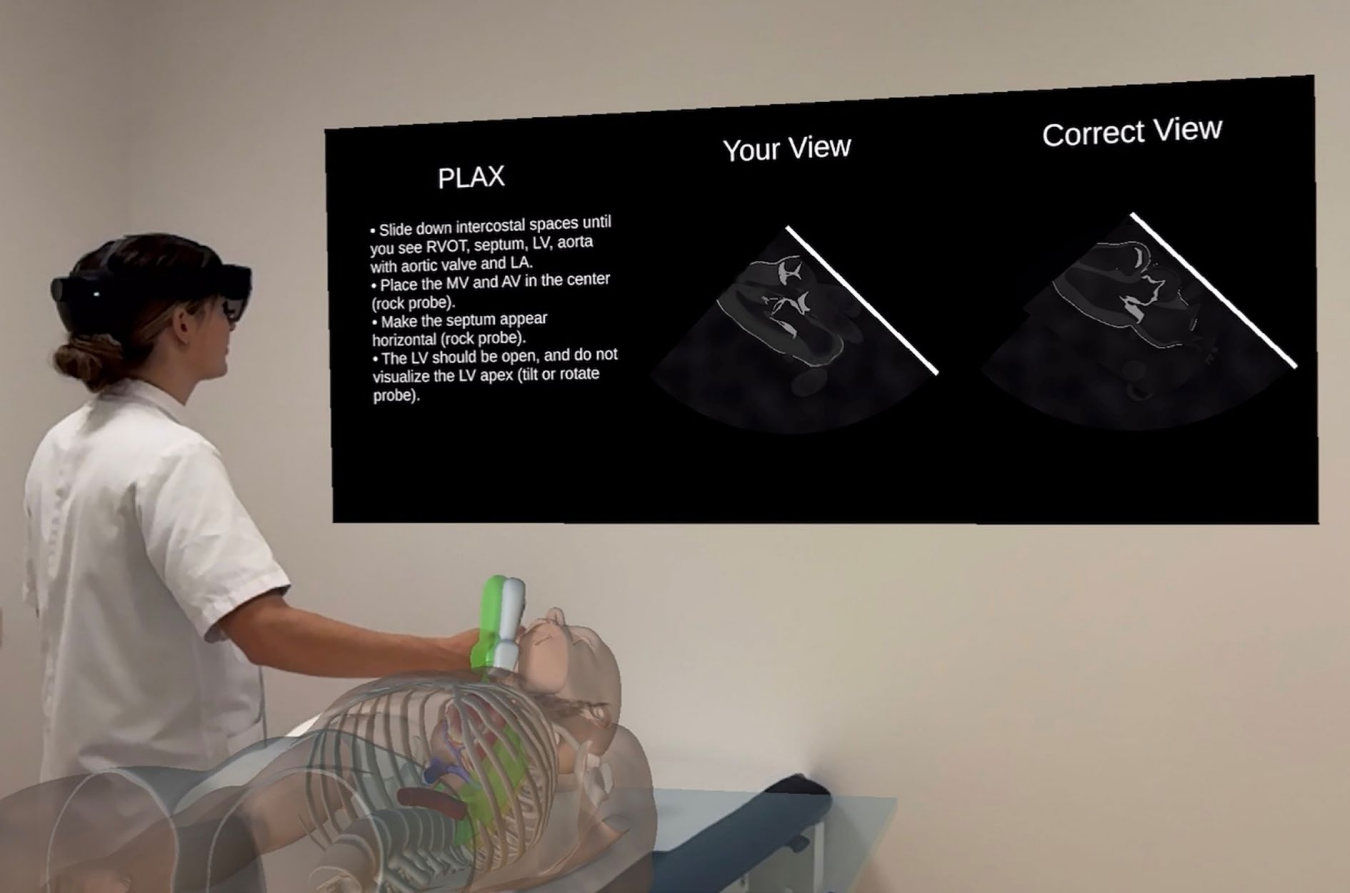
Set‐up of augmedicine: ultrasound in the training phase. The user wears the HoloLens 2 headset, with a phone acting as input device and ultrasound probe. Textual guidance appears in the top left, the user's ultrasound view in the top‐center, and the correct image in the top‐right. A transparent virtual 3D patient model overlays the 3D‐printed phantom, displaying a 3D heart with a white probe indicating the user's position and a green probe showing the correct placement, both illustrating the intersection of ultrasound waves with the model.

Guidance is provided through textual instructions displayed on the left side of the virtual ultrasound screen and a reference image on the right, illustrating the correct ultrasound view. A virtual green probe positioned on the patient further assists users in achieving correct probe placement. Additionally, the phone user interface allows users to toggle through anatomical layers of the 3D model, enabling the user to explore the heart structures. A 3D‐printed thorax phantom of our 3D human model provides haptic feedback to simulate the tactile experience of scanning. The 3D model is projected onto the phantom. Its location is tracked and synchronised with the virtual environment (Figure 1).

#### 2.1.2 Development

The design team included clinical teachers, anatomy experts, ultrasonography experts, developers, educational experts, and a medical illustrator. Our goal was to develop an application that serves a dual purpose. Firstly, it supports ultrasound training by providing an interactive learning experience where users can visualise how ultrasound waves intersect with the heart to produce ultrasound images. This allows users to practise independently, without requiring an ultrasound trainer or a volunteer patient, in an enriched digital learning environment. Secondly, the application is designed to support and study the learning process by collecting objective performance measurements during use, with the possibility to give that back to the user as personalised feedback.

#### 2.1.3 Ultrasound Endpoints

**FIGURE 2:**
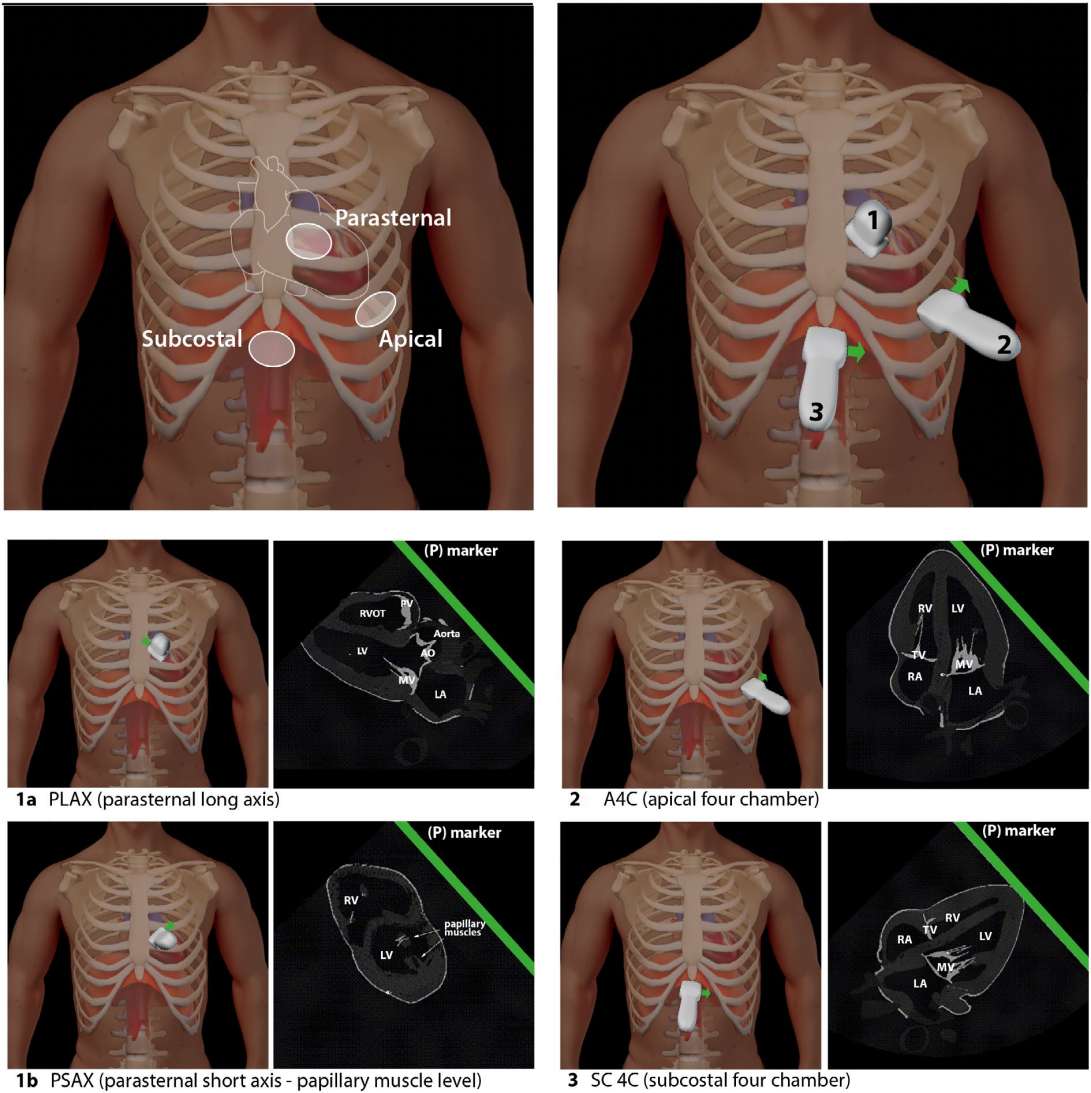
The figure displays the correct ultrasound images. The top left and right images show the probe placements on the thorax, numbered to correspond with the ultrasound images displayed below. The views are numbered and labelled as follows: (1a) parasternal long axis (PLAX), (1b) parasternal short axis (PSAX), (2) apical four‐chamber (A4C), and (3) subcostal four‐chamber (SC4AC).

The app is designed to teach the basic principles of point‐of‐care echocardiography, with a focus on obtaining four essential cardiac views: the parasternal long axis, parasternal short axis, apical 4‐chamber view, and subcostal 4‐chamber view (Figure 2). These views are fundamental for echocardiographic evaluation [14, 15]. The application is modular and can easily be extended to other regions/systems of the human body.

To reach these endpoints, the app is divided into three sequential phases, which can be selected from a menu on the accompanying phone application:

1. Tutorial phase: Users receive step‐by‐step instructions on how to operate the application and interact with the ultrasound simulation. This phase ensures users are familiar with the tools and features.
2. Training phase: Visual guides assist users in placing the probe correctly to obtain the targeted ultrasound views. During this phase, users can explore the 3D heart model by toggling anatomical layers, adjusting transparency, and physically moving around the model to understand spatial relationships.
3. Evaluation phase: Visual guidance is removed, except for the real‐time ultrasound image, and users are challenged to align the probe as accurately and efficiently as possible to obtain the correct ultrasound image. In this phase, one of the four essential views is randomly selected for users to replicate, testing their understanding and skill in probe positioning.

Further details on the technical aspects of the application, along with a video demonstrating its features and functionality, are available as an online supplement (Supporting Information Files 1 and 2 in Appendix S1; Video 1).

**VIDEO 1:** Demonstration of the features and functionality of Augmedicine: Ultrasound. Video content can be viewed at https://onlinelibrary.wiley.com/doi/10.1002/ajum.70019.

### 2.2 Face and Content Validation

Face validity was evaluated by assessing the realism of the application in replicating authentic ultrasound tasks, including the anatomical accuracy of the 3D heart model and the interaction between the virtual probe and simulated ultrasound images. Content validity focused on the application's ability to support learning objectives relevant to ultrasound training, such as improving spatial understanding, probe handling, and interpretation of cross‐sectional anatomy. Stakeholder participants rated these aspects through a structured questionnaire featuring 5‐point Likert‐scale questions (1 = strongly disagree, 2 = disagree, 3 = neutral, 4 = agree, 5 = strongly agree) and open‐ended prompts that were based on previously used questionnaires [6, 14]. These questionnaires can be found in Supporting Information Files 3a‘Questionnaire for novices’ (Appendix S2) and 3b ‘Questionnaire for intermediates and experts’ (Appendix S3).

### 2.3 Participants

Novices, intermediates, and experts in ultrasonography were asked to participate. Novices were medical students in their internal medicine clerkship with minimal to no prior experience in echocardiography, defined as having at most one prior exposure to performing cardiac ultrasound. Intermediates were internists or internal medicine residents at Leiden University Medical Center who had previously followed a 3‐day national ultrasound course but who had performed less than 25 cardiac ultrasounds. Experts were ultrasonography teachers who had performed more than 100 cardiac ultrasounds. None of the participants were involved in the development process of the application. All participants were asked to participate between March 2024 and September 2024 and provided written informed consent. The study was conducted according to the principles of the Declaration of Helsinki (2 October 2013) and the General Data Protection Regulation (GDPR). The protocol of this study was approved by the Educational Research Review Board of the LUMC on 29‐4‐2024 (reference number: OEC/ERRB/20240213/1A).

### 2.4 Study Procedure

Novices underwent ultrasonography training, including a 1‐h lecture on basic cardiac ultrasonography, followed by supervised practice on a volunteer in small groups, providing each participant 20 min of hands‐on experience before training with the application. All participants followed a 10‐min e‐learning with an explanation about the application. Consecutively, they followed the tutorial, training, and evaluation modes of the application and completed the questionnaire mentioned under the subheading ‘face and content validation’.

### 2.5 Statistical Analysis

We used descriptive statistics to describe the answers to the five‐point Likert‐scale questions (percentages). Answers to open‐ended questions were thematically analysed and counted based on visible patterns, primarily to illustrate the answers, and were not formally coded. All analyses were performed in R studio version 2022.02.1 (Posit).

## 3 Results

**FIGURE 3:**
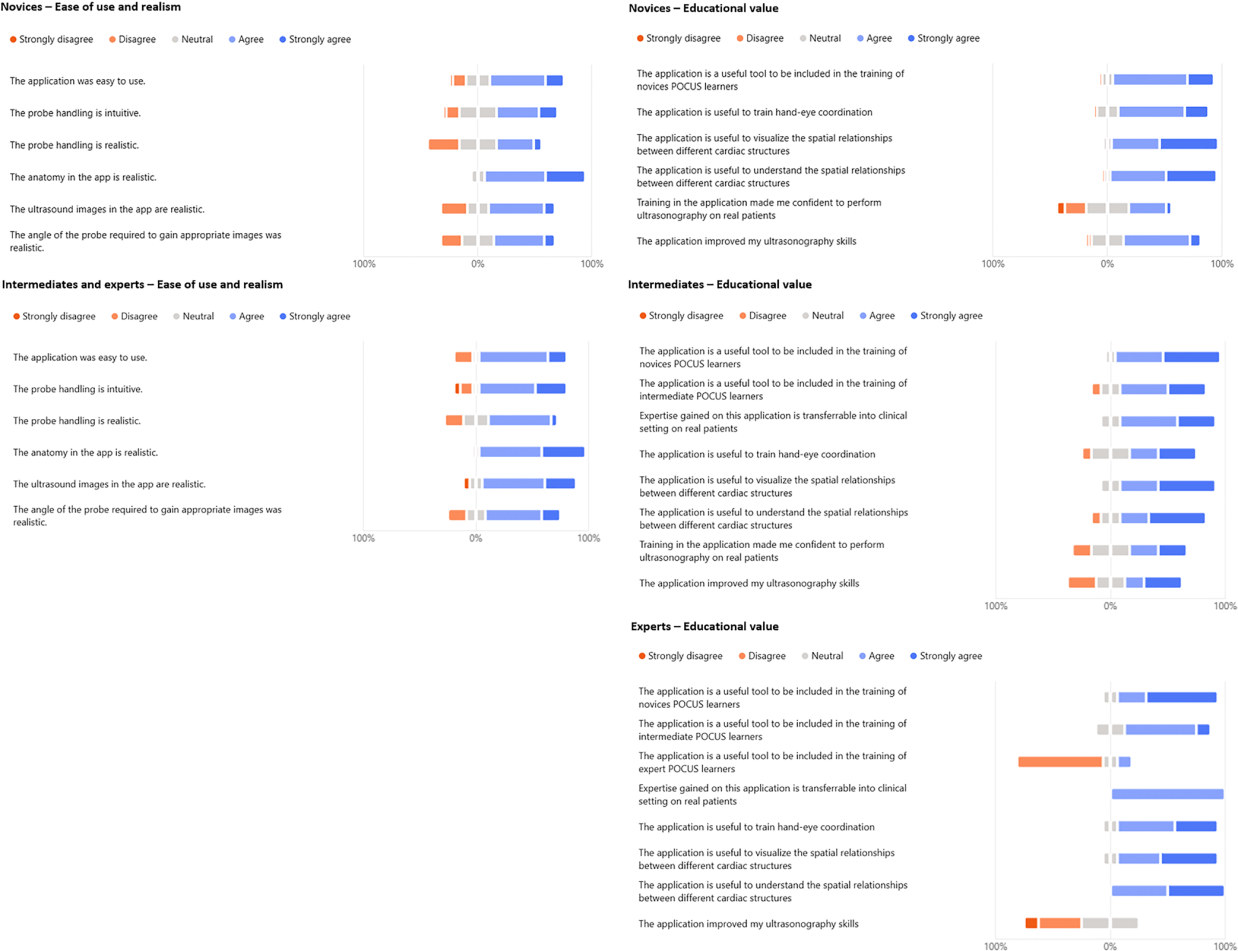
Answers to the Likert‐scale questions (strongly disagree, disagree, neutral, agree, strongly agree) are displayed as percentages of responses given by the group (novices, intermediate or experts).

A total of 63 participants evaluated the application, including 45 novices, 10 intermediates, and 8 experts. Two novices did not complete the questionnaire after training. Participant characteristics are shown in Table 1. Quantitative feedback from the Likert‐scale questions indicated overall positive responses regarding usability, realism, and educational value (Figure 3). A summary of the answers to the open‐ended questions can be found in Table 2.

**TABLE 1:** Characteristics of the participants.

	Novices (*N* = 43)	Intermediates (*N* = 10)	Experts (*N* = 8)
Baseline characteristics			
Age, years	24 ± 1	33 ± 2	35 ± 3
Gender			
Female	38	4	4
Male	15	6	4
Do not want to disclose	—	—	
Level of education			
Medical student	43	—	—
Resident internal medicine	—	9	4
Internist	—	1	2
Emergency physician	—	—	1
Cardiologist	—	—	1
Hand dominance			
Right‐handed	40	10	10
Left‐handed	2	—	—
Gaming experience			
Yes	16	4	2
Hours p/month, median (IQR)	17 (8;40)	10 (8;10)	—
Experience with XR			
No	2	7	5
Yes, < 5 times	37	3	3
Yes, > 5 times	4	—	—
Experience with ultrasound of the heart			
No	37	—	—
Yes, 1 time	6	—	—
Yes, 1–5 times	—	1	—
Yes, 5–25 times	—	9	—
Yes, > 100 times	—	—	8

*Note:* Data are presented in number or mean ± SD unless otherwise indicated.

Abbreviation: XR, eXtended reality.

**TABLE 2:** Answers to the open‐ended questions clustered per category and theme from all 61 participants. Answers divided by experience level can be found in Supporting Informational File 4 (Tables S1–S3).

Category	Questions and answers
Ease of use	**Which technical challenges did you encounter while using the application?**
	Calibration issues (*n* = 32) Handling of the phone as a probe (*n* = 3)
Realism	**Which specific elements of the AR experience did you find particularly convincing?**
	The anatomy and/or ultrasound cross‐sections (*n* = 28) The realistic feedback from probe movements and changes in the ultrasound image (*n* = 8)
	**Which specific elements of the AR experience did you find particularly lacking in realism?**
	The virtual ultrasound images were different from real ultrasound images (*n* = 15) Calibration issues between the phone and the probe/3D model (*n* = 16) The phone as a probe does not feel realistic (*n* = 10)
Educational value	**What aspects of the AR application did you find most beneficial for learning cardiac ultrasonography?**
	To see how the ultrasound waves create a cross‐section through the 3D model and how this translates into the ultrasound image (*n* = 33) The interactive 3D model to clearly see and understand the heart's 3D anatomy and its spatial relationships (*n* = 16) To compare the correct probe position to the user's probe position and receive feedback on how adjustments impact the echocardiographic image (*n* = 13) The opportunity to practice in a calm setting without a volunteer or patient, with the ability to repeat and explore different probe positions and angles (*n* = 6)
	**How do you envision the AR application complementing or integrating with traditional methods of teaching cardiac ultrasonography?**
	As an additional tool or complement to traditional teaching (*n* = 22) A useful first step before practicing on real patients (*n* = 22) To improve anatomy understanding and spatial insight (*n* = 12) As a low‐pressure or independent learning environment (*n* = 7)
	**Are there specific scenarios or topics where you believe the AR approach could be particularly beneficial?**
	As a first step or beginner tool (*n* = 13) For improving anatomy and spatial understanding (*n* = 9) When you do not want to burden volunteers or volunteers are not available (*n* = 11) For procedures and pathology/patient scenarios (*n* = 9) When teaching large groups (*n* = 2) In general ultrasound courses when including other organs (*n* = 2)
	**How do you think the AR application contributed to your understanding of cardiac ultrasonography compared to training on volunteers?**
	Easier to understand anatomy and spatial relationships (*n* = 13) Easier to understand correct probe positioning in relation to anatomy (*n* = 18) As additional practice where you can take your time without a patient or volunteer (*n* = 6)
Improvements	**What additional features would you like to see incorporated into the AR application to enhance your learning experience?**
	Extra options to visualise more of the anatomy (*n* = 4) Including pathology/patient scenarios (*n* = 8) A real probe instead of a phone (*n* = 5) More voice commands for certain menu items (*n* = 2) Extend to more organs to perform ultrasound on (*n* = 1)
	**Are there any specific aspects of the application that you think could be improved to better meet your educational needs?**
	Add more diverse training options (e.g., start without visual aids or give auditive instructions) (*n* = 14) The calibration issues (*n* = 8) Add more features for spatial understanding of heart structures (*n* = 2) Add explanation on how to interpret ultrasound view (*n* = 1) Enhance haptic feedback (make stomach compressible) (*n* = 1)
General feedback	**Do you have any additional comments, thoughts, or feedback about your overall experience?**
	Nice and/or fun learning experience (*n* = 39) This is the future of medical education! (*n* = 3) Unfortunately, I got nauseous (*n* = 1)

*Note:* The total *n* does not always correspond to the number of respondents, as some answers fall into multiple answer themes, while others are not included because they are irrelevant, one‐time occurrences, or left blank.

Abbreviation: *n*, number of times the answer is given.

### 3.1 General Feedback

The application was positively received as a training tool. Most participants found it a useful, fun, and engaging learning experience. One novice stated: ‘It was exactly what I thought I needed when I first tried ultrasound imaging (“I wish my patient were see‐through so I could see where I am aiming”)’. Intermediates highlighted its value in reinforcing their skills without needing a supervisor. An expert said, ‘It is very enjoyable to do, which makes it extra motivating to include in an ultrasound course’.

### 3.2 Ease of Use and Realism

Sixty‐nine percent (42/61) of the participants found the application easy to use (Likert score 4 or 5), indicating an overall good usability (Figure 3). However, 23 (38%) participants noted technical challenges, including calibration delays and tracking inaccuracies (Table 2). These issues occasionally disrupted the overall user experience but did not detract from the application's potential as a training tool. The application was positively rated by 90% of participants for anatomical accuracy, and 28 (45%) of participants specifically highlighted the realism of the anatomy in relation to the ultrasound cross‐sections (Table 2 and Figure 3). Intermediates and experts acknowledged the anatomical realism of the 3D heart model with the ultrasound cross‐sections, but highlighted technical limitations such as occasional lag and the use of a phone as a simulated probe, which detracted from the realism of the simulation. These issues were seen as areas for potential improvement. Additionally, when asked about aspects lacking realism, 15 (25%) participants noted that the ultrasound images differed from those encountered in real‐life settings. However, 40 (66%) participants still found the generated/simulated ultrasound images to be realistic, as shown in Figure 3.

### 3.3 Educational Value

The application received high ratings for its educational value, with 95% of participants rating it positively for enhancing spatial understanding and 77% for improving hand–eye coordination (Figure 3). Fifty‐four percent of participants praised its ability to visualise the cross‐sectional beam of ultrasound waves within the 3D model, clearly linking probe positioning to anatomical structures (Table 2). The application was found to be particularly useful for training novices in preclinical settings, helping them develop foundational skills like probe positioning and cross‐sectional anatomy understanding. And although the simulated ultrasound images were different from real ultrasound images, one novice mentioned, ‘The ultrasound images are indeed different from real life, but that actually helps with understanding anatomy and recognizing structures’. Most novices (70%) thought their ultrasound skills improved. Thirty‐seven percent of the novices reported increased confidence in performing ultrasonography on real patients. Intermediate partipants appreciated the opportunity to consolidate their understanding of probe positioning and cardiac anatomy and the ability to remove structures, such as skin and bones, to better observe cross‐sectional views. An intermediate participant described this as ‘a unique opportunity to observe the cross‐sectional view as it traverses the heart’. Experts found the application less suited for their advanced level of expertise, but highlighted its value for teaching novices and intermediates, particularly in illustrating why certain probe movements are necessary. As one expert stated, ‘It makes it really visual for novice users why they have to move the probe in a different direction’.

### 3.4 Suggested Improvements

Participants proposed several enhancements to improve the educational value of the application. Key suggestions for extended features included the addition of pathology‐specific cases and integrating advanced anatomical features, such as labelled structures or a semi‐transparent myocardium in the 3D model, to enhance visualisation and understanding. Improved features focused on addressing the technical and functional aspects: the phone‐based probe was frequently identified as a limitation, with participants recommending a more realistic device that better mimics the tactile experience of using an actual ultrasound probe. Addressing technical challenges, including lag and calibration inaccuracies, was also highlighted as essential for improving the application's overall fluidity and user experience.

### 3.5 Cybersickness

Moderate cybersickness complaints were reported by six novices, one intermediate, and one expert. Four novices reported severe issues that could be related to cybersickness (fatigue, headache, stomach awareness), though one had pre‐existing stomach complaints. One intermediate had issues that could well be related to cybersickness, with severe complaints of dizziness, eyestrain, and fullness of the head, which resolved swiftly after removing the headset.

## 4 Discussion

We developed an XR application to enhance POCUS training and evaluated its face and content validity to ensure its realism and educational applicability. Face validity refers to the perceived realism and usability of the application, while content validity assesses how well the application aligns with educational objectives and effectively addresses key learning goals. Users of various experience levels responded positively, highlighting the potential for medical education and clinical practice. This study not only demonstrates the application's face and content validity, but also underscores its value in addressing key challenges in POCUS training. The enthusiastic feedback reflects broad support for its use and further development in medical education.

Our choice to use XR technology, and especially AR, was driven by its ability to create an authentic learning environment that enables the projection of a 3D anatomical model onto a phantom, providing users with crucial haptic feedback. Additionally, AR allows the same 3D projection to be overlaid onto a real person, such as a volunteer or patient, providing a seamless transition from simulated to real‐life practice. Unlike VR applications, where cybersickness is a common concern, we observed minimal instances of cybersickness, probably because AR keeps users grounded in their physical surroundings [15]. Furthermore, the integration of 3D anatomical visualisation with interactive features links probe positioning to cross‐sectional images, improving spatial understanding and bridging the gap between theory and practice. This approach aligns with the broader trend of adopting XR tools in medical education, offering scalable solutions to overcome the scarcity of trainers and training resources [5, 16]. All groups highlighted that the use of the AR application could support skill development and build confidence early in training. While the application received overall positive feedback, participants identified areas for improvement. The use of a phone as a probe was a recurrent concern, with participants noting that it detracted from realism. Technical challenges such as calibration delays and synchronisation issues were also reported. Addressing these limitations, particularly by incorporating a more realistic probe and optimising tracking accuracy, would further enhance the realism and educational value of the application. One could think about tracking a real ultrasound probe or, e.g., use generic XR controllers, although the latter may evoke similar concerns for realism.

The XR application is particularly valuable in preparing novice learners for clinical scenarios, who often struggle with understanding spatial relationships as well as developing hand–eye coordination and psychomotor skills for probe handling—key aspects for acquiring POCUS competence [4, 17]. As participants highlighted, the application can serve as a supplementary tool that replaces certain aspects of the current hands‐on practice, enhancing spatial understanding and self‐efficacy, and facilitating the transition to patient‐based practice. Part of the effectiveness of the XR application lies in its ability to provide 3D visualisation of anatomy, which has been shown in prior research to support competence development [9]. By allowing learners to interact with holograms in a truly 3D space, the application enhances depth perception and offers a more realistic representation of anatomy. This approach further aids spatial orientation and improves understanding of complex anatomical structures [18]. Evidence of these benefits is seen in similar XR applications; for example, Hu et al. demonstrated that VR‐enhanced anatomical training significantly improved ultrasound competence, and Khoo et al. recently showed the effectiveness of their VR‐based echocardiography training [8, 19]. While intermediate and expert participants noted the potential for the application to build novices' confidence prior to patient practice, novices themselves reported improved technical skills but no corresponding increase in confidence for performing ultrasound on real patients. This hesitancy likely reflects the intimidating nature of patient contact for medical students, underscoring the need for a structured transition from simulation to clinical practice [20].

Beyond novice training, the application holds promise for lifelong learning and professional development. As POCUS becomes increasingly integrated into diverse medical specialities, there is a growing need to provide ongoing training and skill development for professionals who have mastered the basics [4]. XR technology could play a crucial role in this shift by providing accessible, scalable training solutions for clinicians at different stages of their careers. The addition of pathologies or other clinical scenarios could well motivate more experienced stakeholders to develop extra competence in a well‐accessible training environment.

While the findings of this study are promising to integrate the application in POCUS education, it should be noted that the study population may not be fully representative due to potential selection bias. Participants who voluntarily took part may have had a pre‐existing interest in ultrasound or XR technologies, which could have positively influenced their evaluations. Conversely, there may be learners with less affinity for ultrasound or XR who find the application less intuitive or engaging, potentially limiting its effectiveness for this group.

In conclusion, this study demonstrates that the XR application for echocardiography training meets face and content validity criteria, offering notable benefits, particularly for novice learners. By providing 3D anatomical guidance, realistic probe movements, and an engaging learning environment, the application could complement traditional ultrasound training. It achieves this by improving spatial anatomical understanding and reducing the need for human resources such as instructors and volunteers during the initial training phases, and by being self‐contained and easily accessible. However, improving and expanding the application is an iterative process that relies on regular feedback and evaluation. Future research should focus on the application's effectiveness for learning ultrasonography, while addressing identified limitations, such as the use of a phone as a probe and calibration challenges, to further enhance the application's realism and usability.

## Author Contributions

T.A.M. conceived the study, coordinated all stages of the project, and was responsible for data collection, analysis, and drafting and revising the manuscript. R.J. contributed to participant inclusion, data collection and manuscript drafting. A.Q. and M.S.Z. were primarily involved in the development of the XR application and contributed to reviewing and revising the manuscript. M.P.B., A.M.J.L., A.S. and B.P.H. contributed to the design of the study, interpretation of the results, and critical revision of the manuscript. All authors read and approved the final manuscript.

## Conflicts of Interest

The authors of this article are also the developers of the XR application evaluated in this study. While this dual role allowed for detailed insights into the application's functionality and design, it may introduce potential bias in the interpretation of results. Efforts were made to minimise this bias by employing validated assessment methods and involving independent experts in the evaluation process. The authors have no commercial interests related to the XR application.

## Supporting information


**Appendix S1:** ajum70019‐sup‐0001‐AppendixS1.docx.

**Appendix S2:** ajum70019‐sup‐0002‐AppendixS2.pdf.

**Appendix S3:** ajum70019‐sup‐0003‐AppendixS3.pdf.

Table S1‐S3: S1–S3
