# Supplementary material for: Development and Face and Content Validation of Augmedicine: Ultrasound—An Extended Reality Application for Ultrasound Training
Source: Australas J Ultrasound Med. 2025 Aug 21;28(3):e70019. doi: 10.1002/ajum.70019 (PMC12368875; doi:10.1002/ajum.70019)
Supplement: Supplementary file 1 [file AJUM-28-0-s003.pdf]

Questionnaire for novices

1. What is your study ID? *

2. Application rating *

	Strongly disagree	Disagree	Neutral	Agree
The application was easy to use.	○	○	○	○
The probe handling is intuitive.	○	○	○	○
The probe handling is realistic.	○	○	○	○
The anatomy in the app is realistic.	○	○	○	○
The ultrasound images in the app are realistic.	○	○	○	○
The angle of the probe required to gain appropriate images was realistic.	○	○	○	○

3. Didactive value *

	Strongly disagree	Disagree	Neutral	Agree
The application is a useful tool to be included in the training of novices POCUS learners				
The application is useful to train hand-eye coordination				
The application is useful to visualize the spatial relationships between different cardiac structures				
The application is useful to understand the spatial relationships between different cardiac structures				
Training in the application made me confident to perform ultrasonography on real patients				
The application improved my ultrasonography skills				

4. ARSQ; Place a cross next to the number indicating the degree of your experienced symptoms for each item.
0 = none, 1 = Slight, 2 = Moderate, 3 = Severe.
*

	0	1	2
Is there any general discomfort?	○	○	○
Do you feel fatigued?	○	○	○
Do you have a headache?	○	○	○
Do you feel eye strain?	○	○	○
Do you feel the fullness of your head?	○	○	○
Do you feel dizzy with your eyes open?	○	○	○
Do you feel dizzy with your eyes closed?	○	○	○
Do you feel vertigo?	○	○	○
Do you feel any stomach awareness?	○	○	○

Open-ended questions

5. What aspects of the augmented reality (AR) application did you find most beneficial for learning cardiac ultrasonography? *

6. Which specific elements of the AR experience you found particularly lacking in realism? *

7. Which specific elements of the AR experience you found particularly convincing? *

8. Which technical challenges did you encounter while using the application? *

9. What additional features would you like to see incorporated into the AR application to enhance your learning experience? *

10. Are there any specific aspects of the application that you think could be improved to better meet your educational needs? *

11. How do you envision the AR application complementing or integrating with traditional methods of teaching cardiac ultrasonography? *

12. How do you think the AR application contributed to your understanding of cardiac ultrasonography compared to training on volunteers? *

13. Are there specific scenarios or topics where you believe the AR approach could be particularly beneficial? *

14. Do you have any additional comments, thoughts, or feedback about your overall experience with the AR application for heart ultrasonography training? *
