# Supplementary material for: Development and Face and Content Validation of Augmedicine: Ultrasound—An Extended Reality Application for Ultrasound Training
Source: Australas J Ultrasound Med. 2025 Aug 21;28(3):e70019. doi: 10.1002/ajum.70019 (PMC12368875; doi:10.1002/ajum.70019)
Supplement: Supplementary file 2 [file AJUM-28-0-s004.pdf]

# Questionnaire for intermediates and experts

1. Age *

2. Gender *

- Male
- Female
- Other
- Do not want to disclose

3. Level op education *

- Resident internal medicine
- Internist
- Cardiologist
- Andere

4. Hand dominance *

- Left-handed
- Right-handed

5. Gaming experience *

- Yes
- No

6. If yes, estimation of number of hours per month *

7. Prior experience with AR/VR *

- Yes, < 5 times
- Yes, > 5 times
- No

8. Prior experience with performing ultrasound *

- < 5 times
- 5 - 25 times
- 25 - 50 times
- 50 - 100 times
- > 100 times

9. Prior experience with performing ultrasound of the heart *

- ◯ No
- ◯ 1 - 5 times
- ◯ 5 -25 times
- ◯ 25 - 50 times
- ◯ 50 - 100 times
- ◯ > 100 times

10. Application rating

| | Strongly disagree | Disagree | Neutral | Agree |
|---|---|---|---|---|
| The application was easy to use. | ◯ | ◯ | ◯ | ◯ |
| The probe handling is intuitive. | ◯ | ◯ | ◯ | ◯ |
| The probe handling is realistic. | ◯ | ◯ | ◯ | ◯ |
| The anatomy in the app is realistic. | ◯ | ◯ | ◯ | ◯ |
| The ultrasound images in the app are realistic. | ◯ | ◯ | ◯ | ◯ |
| The angle of the probe required to gain appropriate images was realistic. | ◯ | ◯ | ◯ | ◯ |

11. Are you an intermediate or expert? *

◯ Intermediate

◯ Expert

12. Didactive value (Experts) *

| | Strongly disagree | Disagree | Neutral | Agree |
|---|---|---|---|---|
| The application is a useful tool to be included in the training of novices POCUS learners | ◯ | ◯ | ◯ | ◯ |
| The application is a useful tool to be included in the training of intermediate POCUS learners | ◯ | ◯ | ◯ | ◯ |
| The application is a useful tool to be included in the training of expert POCUS learners | ◯ | ◯ | ◯ | ◯ |
| Expertise gained on this application is transferrable into clinical setting on real patients | ◯ | ◯ | ◯ | ◯ |
| The application is useful to train hand-eye coordination | ◯ | ◯ | ◯ | ◯ |
| The application is useful to visualize the spatial relationships between different cardiac structures | ◯ | ◯ | ◯ | ◯ |
| The application is useful to understand the spatial relationships between different cardiac structures | ◯ | ◯ | ◯ | ◯ |
| The application improved my ultrasonograph y skills | ◯ | ◯ | ◯ | ◯ |

13. Didactive value (Intermediates) *

| | Strongly disagree | Disagree | Neutral | Agree |
|---|---|---|---|---|
| The application is a useful tool to be included in the training of novices POCUS learners | ◯ | ◯ | ◯ | ◯ |
| The application is a useful tool to be included in the training of intermediate POCUS learners | ◯ | ◯ | ◯ | ◯ |
| Expertise gained on this application is transferrable into clinical setting on real patients* | ◯ | ◯ | ◯ | ◯ |
| The application is useful to train hand-eye coordination | ◯ | ◯ | ◯ | ◯ |
| The application is useful to visualize the spatial relationships between different cardiac structures | ◯ | ◯ | ◯ | ◯ |
| The application is useful to understand the spatial relationships between different cardiac structures | ◯ | ◯ | ◯ | ◯ |
| Training in the application made me confident to perform ultrasonography on real patients | ◯ | ◯ | ◯ | ◯ |
| The application improved my ultrasonography skills | ◯ | ◯ | ◯ | ◯ |

14. ARSQ; Place a cross next to the number indicating the degree of your experienced symptoms for each item.
0 = none, 1 = Slight, 2 = Moderate, 3 = Severe.
*

| | 0 | 1 | 2 |
|---|---|---|---|
| Is there any general discomfort? | ○ | ○ | ○ |
| Do you feel fatigued? | ○ | ○ | ○ |
| Do you have a headache? | ○ | ○ | ○ |
| Do you feel eye strain? | ○ | ○ | ○ |
| Do you feel the fullness of your head? | ○ | ○ | ○ |
| Do you feel dizzy with your eyes open? | ○ | ○ | ○ |
| Do you feel dizzy with your eyes closed? | ○ | ○ | ○ |
| Do you feel vertigo? | ○ | ○ | ○ |
| Do you feel any stomach awareness? | ○ | ○ | ○ |

## Open-ended questions

15. What aspects of the augmented reality (AR) application did you find most beneficial for learning cardiac ultrasonography? *

16. Which specific elements of the AR experience you found particularly convincing? *

17. Which specific elements of the AR experience you found particularly lacking in realism? *

18. Which technical challenges did you encounter while using the application? *

19. What additional features would you like to see incorporated into the AR application to enhance the learning experience? *

20. Are there any specific aspects of the application that you think could be improved to better meet your educational needs? *

21. How do you think the AR application contributed (or could contribute) to your understanding of cardiac ultrasonography compared to training on volunteers? *

22. How do you envision the AR application complementing or integrating with traditional methods of teaching cardiac ultrasonography? *

23. Are there specific scenarios or topics where you believe the AR approach could be particularly beneficial? *

24. Do you have any additional comments, thoughts, or feedback about your overall experience with the AR application for heart ultrasonography training? *
