# Supplementary material for: Development and Face and Content Validation of Augmedicine: Ultrasound—An Extended Reality Application for Ultrasound Training
Source: Australas J Ultrasound Med. 2025 Aug 21;28(3):e70019. doi: 10.1002/ajum.70019 (PMC12368875; doi:10.1002/ajum.70019)
Supplement: Supplementary file 3 [file AJUM-28-0-s001.docx]

**Online supplement. Technical features of Augmedicine Ultrasound**

Technical aspects

The application was developed specifically for use on the Microsoft HoloLens 2. Unity (Unity Technologies, version 2020.3 LTS, <https://unity.com>) served as the foundation for integrating the various technologies needed to bring the application to life.

To create a realistic simulated ultrasound experience, precise tracking between the HoloLens and the virtual ultrasound probe (phone) was essential. This ensured accurate relay of positional and rotation data from the phone to the HoloLens, allowing users to visualize the probe’s movement and the projection of ultrasound waveforms through the 3D heart model. Initially, the idea of attaching a QR code to a probe for positional tracking was explored, but the accuracy was insufficient. Subsequently, a tracking solution based ARKit for iOS and ARCore for Android was developed and tested initially with an Android system (Samsung S20), leveraging the phone’s internal gyroscope and sensors, but the results did not meet the required standards. Ultimately, we used the same technique with an iPhone with LiDAR technology as the simulated ultrasound probe, which provided superior tracking accuracy. For tracking the phone, we employed Vuforia SDK 10.2.5 for calibration between the devices and the phantom and ARFoundation for six degree-of-freedom tracking, which provided compatibility with ARKit. Websockets were implemented to enable real-time communication between the HoloLens and the phone. This networking feature ensured smooth synchronization of data, such as probe position and generated ultrasound images, between the devices. Calibration between the HoloLens and the phone was achieved using a QR code as a shared origin point. For users to interact with the application, we developed a user interface on the smartphone. This interface allows users to select different modes, toggle anatomical parts on and off, and adjust settings (i.e. move virtual ultrasound screen closer) to tailor their training experience.

3D anatomical model

The 3D heart model for the XR application was developed by a medical illustrator in collaboration with clinical and technical experts to create an anatomically accurate, animated heart that supports realistic ultrasound image generation. Using an anonymized CT scan of a healthy heart, detailed analysis in Horos software (Horos Project, Version 4.0.0, <https://horosproject.org>) enabled 3D reconstruction. The resulting raw model was refined in 3D modeling software (Cinema 4D, Version 2022, Maxon, <https://www.maxon.net>), where heart chambers, valves, and blood flow pathways were reconstructed (online figure 1.)

**Online figure 1. (a) shows a side view of the 'CT-slider,' where the heart is modeled over the CT slices. (b) displays the CT scan with vascular contrast, alongside a 3D reconstruction of the heart in contrast. Additionally, a superimposed 3D reconstruction highlights structures not visible in the CT scan.**

The heart models internal and external surfaces were tagged and used to identify tissue components that were assigned grayscale values via a custom shader, enabling ultrasound image simulation. For the user, the heart model was designed as a visual reference, resembling illustrations in anatomy textbooks (online figure 2).

**Online figure 2. (a) A still frame from the simulated 3D heart model. (b) The 3D human model displays a transparent view of the body, including the skeleton, heart, and diaphragm, providing anatomical context for the heart's placement within the thorax.**

To animate the model, Blender software (Blender Foundation, Version 3.2, [https://www.blender.org](https://www.blender.org/)) was used. Animation timing was guided by existing 3D heart models and cardiac imaging videos. The animated heart was integrated into a digital torso, which was also 3D-printed to provide haptic feedback. A QR code on the printed torso ensured alignment between the digital and physical models (online figure 3).

**Online figure 3. The 3D-printed thorax phantom, created from the 3D human model. A QR code for calibration between the HoloLens and the phone is positioned on the square platform where the head would be. The phantom serves as a haptic component, providing tactile feedback to enhance the simulation experience.**

Ultrasound images

Generating realistic ultrasound images from the 3D heart model was not feasible using existing online resources, so the team opted for a novel approach by developing a custom solution. The custom shader used the five distinct layers of the medically illustrated 3D heart model: the pericardium, blood, myocardium, arteries, and valves. An invisible, thin slice (measuring 25 cm x 0.1 cm) was attached to the tip of the virtual probe and moved dynamically with it as the user adjusted the probe's position. Using a third-party Unity plugin, the algorithm sliced through the meshes of each heart layer individually, based on the slice’s position and rotation relative to the heart. A virtual camera then captured images of the intersection areas created by the slicing process for each layer. These images were subsequently overlaid, with each layer represented in a color that corresponded to its real-world ultrasound counterpart. To enhance realism, noise was added to the composite image, and a cone-shaped mask was applied. The resulting ultrasound-like image was then displayed on the virtual screen for the user. These images were dynamically generated in real time, as we are working with a beating heart. Ultrasonography experts selected the four correct echocardiographic views: the parasternal long axis (PLAX), parasternal short axis (PSAX), apical 4-chamber view (4AC), and subcostal 4-chamber view (SC4AC). The app displays the user's image alongside the expert's image on a virtual ultrasound screen, allowing for direct comparison to the correct ultrasound view. Simultaneously, the correct probe position is visualized in green, with the ultrasound beam extending from the probe, allowing comparison with the user's probe position. See online figure 4.

**Online figure 4. User training with the application in the emergency department. The top-left panel displays the ultrasound screen with textual guidance on performing the apical 4-chamber (A4C) view. To the right, the user’s ultrasound view (‘your view’) is shown alongside the correct view for comparison. In the middle section, the 3D model projected onto the phantom displays the skeleton and heart. The correct probe position is visualized in green, with a corresponding green ultrasound beam extending from it. The user’s probe position is visualized in white, with a yellow beam extending, enabling a direct comparison between the two positions.**
