# Supplementary material for: Development and Face and Content Validation of Augmedicine: Ultrasound—An Extended Reality Application for Ultrasound Training
Source: Australas J Ultrasound Med. 2025 Aug 21;28(3):e70019. doi: 10.1002/ajum.70019 (PMC12368875; doi:10.1002/ajum.70019)
Supplement: Supplementary file 4 [file AJUM-28-0-s002.docx]

**Online Table 1. Answers to the open-ended questions clustered per category and theme from novices**

| *Category* | *Questions and answers* |
| --- | --- |
| *Ease of use* | **Which technical challenges did you encounter while using the application?** |
|  | - Calibration issues (n= 20) |
|  | - Handling of the phone as a probe (n=3) |
|  |  |
| *Realism* | **Which specific elements of the AR experience did you find particularly convincing?** |
|  | - The anatomy and/or ultrasound cross-sections (n=12) |
|  | - The realistic feedback from probe movements and changes in the ultrasound   image (n= 8) |
|  |  |
|  | **Which specific elements of the AR experience did you find particularly lacking in realism?** |
|  | - The virtual ultrasound images were different from real ultrasound images   (n=12) |
|  | - Calibration issues between the phone and the probe/3D model (n= 11) |
|  | - The phone as a probe doesn’t feel realistic (n=7) |
|  |  |
| *Educational Value* | **What aspects of the AR application did you find most beneficial for learning cardiac ultrasonography?** |
|  | - To see how the ultrasound waves create a cross-section through the 3D model   and how this translates into the ultrasound image (n = 20) |
|  | - The interactive 3D model to clearly see and understand the heart's 3D anatomy   and its spatial relationships. (n = 16) |
|  | - To compare the correct probe position to the user's probe position and receive   feedback on how adjustments impact the echocardiographic image. (n = 9) |
|  | - The opportunity to practice in a calm setting without a volunteer or patient,   with the ability to repeat and explore different probe positions and angles. (n =  4) |
|  |  |
|  | **How do you envision the AR application complementing or integrating with traditional methods of teaching cardiac ultrasonography?** |
|  | - As an additional tool or complement to traditional teaching (n = 16) |
|  | - A useful first step before practicing on real patients (n = 12) |
|  | - To improve anatomy understanding and spatial insight (n = 9) |
|  | - As a low-pressure or independent learning environment (n = 7) |
|  |  |
|  | **Are there specific scenarios or topics where you believe the AR approach could be particularly beneficial?** |
|  | - As a first step or beginner tool (n=8) |
|  | - For improving anatomy and spatial understanding (n=7) |
|  | - When you don’t want to burden volunteers or volunteers are not   available (n=11) |
|  | - Practicing abnormal or complex cases (n = 5) |
|  | - When teaching large groups (n=3) |
|  |  |
|  | **How do you think the AR application contributed to your understanding of cardiac ultrasonography compared to training on volunteers?** |
|  | - Easier to understand anatomy and spatial relationships (n = 9) |
|  | - Easier to understand correct probe positioning (n=7) |
|  | - As additional practice where you can take your time without a patient or   volunteer (n = 4) |
|  |  |
| *Improvements* | **What additional features would you like to see incorporated into the AR application to enhance your learning experience?** |
|  | - Extra options to visualize more of the anatomy (n=4) |
|  | - Including pathology (n=4) |
|  | - A real probe instead of a phone (n=3) |
|  |  |
|  | **Are there any specific aspects of the application that you think could be improved to better meet your educational needs?** |
|  | - Add more diverse training options (e.g. start without visual aids or give auditive   instructions) (n=6) |
|  | - The calibration issues (n=5) |
|  | - Add more features for spatial understanding of heart structures (n=2) |
|  |  |
| *General Feedback* | **Do you have any additional comments, thoughts, or feedback about your overall experience?** |
|  | - Very nice and/or fun learning experience (n=17) |

**n= number of times the answer is given. Note: The total n does not always correspond to the number of respondents, as some answers fall into multiple answer themes, while others are not included because they are irrelevant, one-time occurrences, or left blank**

**Online Table 2. Answers to the open-ended questions clustered per category and theme from intermediates**

| *Category* | *Questions and answers* |
| --- | --- |
| *Ease of use* | **Which technical challenges did you encounter while using the application?** |
|  | - Calibration issues (n= 7) |
|  | - The holograms were not visible enough (n=1) |
|  | - No major challenges (n=1) |
|  |  |
| *Realism* | **Which specific elements of the AR experience did you find particularly convincing?** |
|  | - The anatomy in relation to the probe positions / ultrasound images   (n=8) |
|  | - The anatomy (n= 2) |
|  |  |
|  | **Which specific elements of the AR experience did you find particularly lacking in realism?** |
|  | - The ultrasound images were differed from real images (n=2) |
|  | - Calibration issues between the phone and the probe/3D model (n= 2) |
|  | - The phone as a probe doesn’t feel realistic (n=1) |
|  |  |
| *Educational Value* | **What aspects of the AR application did you find most beneficial for learning cardiac ultrasonography?** |
|  | - To see how the ultrasound waves create a cross-section through the 3D   model and how this translates into the ultrasound image (n = 6) |
|  | - To compare the correct probe position to the user's probe position   (n=4) |
|  | - The interactive 3D model. (n=2) |
|  |  |
|  | **How do you envision the AR application complementing or integrating with traditional methods of teaching cardiac ultrasonography?** |
|  | - As an additional training tool for intermediates to retrain or maintain   skills (n= 5) |
|  | - A useful first step before practicing on real patients (n = 4) |
|  | - To improve anatomy understanding and spatial insight (n = 1) |
|  |  |
|  | **Are there specific scenarios or topics where you believe the AR approach could be particularly beneficial?** |
|  | - As a first step and/or additional training tool (n=2) |
|  | - For improving anatomy and spatial understanding (n=1) |
|  | - In general ultrasound courses when including other organs (n=1) |
|  | - No answer (n=6) |
|  |  |
|  | **How do you think the AR application contributed to your understanding of cardiac ultrasonography compared to training on volunteers?** |
|  | - Easier to understand probe positioning in relation to anatomy (n=7) |
|  | - Easier to understand anatomy and spatial relationships (n = 3) |
|  | - As extra practice time without patient/volunteer (n =2) |
|  |  |
| *Improvements* | **What additional features would you like to see incorporated into the AR application to enhance your learning experience?** |
|  | - More voice commands for certain menu items (n=2) |
|  | - A real probe instead of a phone (n=2) |
|  | - Extend to more organs to perform ultrasound on (n=1) |
|  |  |
|  | **Are there any specific aspects of the application that you think could be improved to better meet your educational needs?** |
|  | - The calibration issues (n=2) |
|  | - Enhance haptic feedback (make stomach compressible) (n=1) |
|  | - A cover for the phone so that it feels more like a probe (n=1) |
|  | - Add explanation on how to interpret ultrasound view (n=1) |
|  |  |
| *General Feedback* | **Do you have any additional comments, thoughts, or feedback about your overall experience?** |
|  | - Very beneficial and/or fun! (n=5) |
|  | - Unfortunately, I got nauseous (n=1) |
|  | - No (n=4) |

**n= number of times the answer is given. Note: The total n does not always correspond to the number of respondents, as some answers fall into multiple answer themes, while others are not included because they are irrelevant, or left blank**

**Online Table 3. Answers to the open-ended questions clustered per category and theme from experts**.

| *Category* | *Question and answers* |
| --- | --- |
| *Ease of use* | **Which technical challenges did you encounter while using the application?** |
|  | - Calibration issues (n= 5) |
|  | - Device had to be restarted (n=2) |
|  |  |
| *Realism* | **Which specific elements of the AR experience you found particularly convincing?** |
|  | - The anatomy in relation to the probe positions / ultrasound images   (n=3) |
|  | - The anatomy (n= 3) |
|  |  |
|  | **Which specific elements of the AR experience you found particularly lacking in realism?** |
|  | - Delay in probe movement (calibration issue) (n= 3) |
|  | - The phone as a probe doesn’t feel realistic (n=2) |
|  | - The ultrasound images were different from real images (n=1) |
|  |  |
| *Educational Value* | **What aspects of the AR application did you find most beneficial for learning cardiac ultrasonography?** |
|  | - To see how the ultrasound waves create a cross-section through the 3D   model and how this translates into the ultrasound image (n = 7) |
|  | - Practice time without patient burden (n=2) |
|  |  |
|  | **How do you envision the AR application complementing or integrating with traditional methods of teaching cardiac ultrasonography?** |
|  | - A useful first step before practicing on real patients (n = 6) |
|  | - To improve anatomy understanding and spatial insight (n = 2) |
|  | - As an additional training tool after or during an ultrasound course to   retrain or maintain skills (n= 1) |
|  |  |
|  | **Are there specific scenarios or topics where you believe the AR approach could be particularly beneficial?** |
|  | - As a training tool for novices (n=3) |
|  | - For procedures and pathology/patient scenarios (n=4) |
|  | - For improving anatomy and spatial understanding (n=1) |
|  | - For remote training (n=1) |
|  |  |
|  | **How do you think the AR application contributed (or could contribute) to your understanding of cardiac ultrasonography compared to training on volunteers?** |
|  | - Easier to understand probe positioning in relation to anatomy for   novices (n=4) |
|  | - Not contributing for expert users (n = 3) |
|  | - To see the 3D anatomical structures (n =1) |
|  |  |
| *Improvements* | **What additional features would you like to see incorporated into the AR application to enhance your learning experience?** |
|  | - A real probe instead of a phone (n=2) |
|  | - Add pathology/patient scenarios (n=2) |
|  | - Add explanation on interpretation of ultrasound images (n=1) |
|  |  |
|  | **Are there any specific aspects of the application that you think could be improved to better meet your educational needs?** |
|  | - The calibration issue in the subcostal view (n=1) |
|  | - More realistic echo probe (n=1) |
|  | - Extended stepwise explanation for probe positioning (n=1) |
|  | - Add pathology (n=1) |
|  |  |
| *General Feedback* | **Do you have any additional comments, thoughts, or feedback about your overall experience?** |
|  | - Nice application/impressive/fun and motivating! (n=6) |
|  | - No (n=2) |
|  |  |

**n= number of times the answer is given. Note: The total n does not always correspond to the number of respondents, as some answers fall into multiple answer themes, while others are not included because they are irrelevant, or left blank**
